# Examining the long-term effects of traumatic brain injury on fear extinction in male rats

**DOI:** 10.3389/fnbeh.2023.1206073

**Published:** 2023-06-16

**Authors:** K. A. Smith, M. R. Raskin, M. H. Donovan, V. Raghunath, S. Mansoorshahi, M. J. Telch, J. Shumake, L. J. Noble-Haeusslein, M. H. Monfils

**Affiliations:** ^1^Department of Psychology, The University of Texas at Austin, Austin, TX, United States; ^2^Department of Neurology, Dell Medical School, The University of Texas at Austin, Austin, TX, United States; ^3^Institute of Mental Health Research, The University of Texas at Austin, Austin, TX, United States

**Keywords:** traumatic brain injury, CO_2_, fear conditioning, extinction, individual differences

## Abstract

There is a strong association between traumatic brain injuries (TBIs) and the development of psychiatric disorders, including post-traumatic stress disorder (PTSD). Exposure-based therapy is a first-line intervention for individuals who suffer from PTSD and other anxiety-related disorders; however, up to 50% of individuals with PTSD do not respond well to this approach. Fear extinction, a core mechanism underlying exposure-based therapy, is a procedure in which a repeated presentation of a conditioned stimulus in the absence of an unconditioned stimulus leads to a decrease in fear expression, and is a useful tool to better understand exposure-based therapy. Identifying predictors of extinction would be useful in developing alternative treatments for the non-responders. We recently found that CO_2_ reactivity predicts extinction phenotypes in rats, likely through the activation of orexin receptors in the lateral hypothalamus. While studies have reported mixed results in extinction of fear after TBI, none have examined the long-term durability of this phenotype in the more chronically injured brain. Here we tested the hypothesis that TBI results in a long-term deficit in fear extinction, and that CO_2_ reactivity would be predictive of this extinction phenotype. Isoflurane-anesthetized adult male rats received TBI (*n* = 59) (produced by a controlled cortical impactor) or sham surgery (*n* = 29). One month post-injury or sham surgery, rats underwent a CO_2_ or air challenge, followed by fear conditioning, extinction, and fear expression testing. TBI rats exposed to CO_2_ (TBI-CO_2_) showed no difference during extinction or fear expression relative to shams exposed to CO_2_ (sham-CO_2_). However, TBI-CO_2_ rats, showed significantly better fear expression than TBI rats exposed to air (TBI-air). In contrast to previous findings, we observed no relationship between CO_2_ reactivity and post-extinction fear expression in either the sham or TBI rats. However, compared to the previously observed naïve sample, we observed more variability in post-extinction fear expression but a very similar distribution of CO_2_ reactivity in the current sample. Isoflurane anesthesia may lead to interoceptive threat habituation, possibly via action on orexin receptors in the lateral hypothalamus, and may interact with CO_2_ exposure, resulting in enhanced extinction. Future work will directly test this possibility.

## 1. Introduction

According to recent data from the Centers for Disease Control and Prevention (CDC), there were approximately 223,135 traumatic brain injury (TBI)-related hospitalizations and 64,362 deaths in 2019 alone (CDC, 2022). Males were twice as likely as females to be hospitalized, with three times the risk of mortality, spanning early life to the aged population (Center for Disease Control and Prevention, 2018-2022). TBIs are well-known for their heterogeneity (Saatman et al., 2008), which is, in part, attributed to the variable nature and severity of the insult and brain regions involved. Regardless of this heterogeneity, there is a strong association between TBIs and the subsequent development of psychiatric disorders, including altered mood, psychoses, anxiety, stress, depression, substance abuse and posttraumatic stress disorders (PTSD) (Whelan-Goodinson et al., 2009; Gould et al., 2011; Koponen et al., 2011; Alway et al., 2016; Ponsford et al., 2018). PTSD frequently presents as a comorbid condition among brain-injured patients (Ponsford et al., 2018); however, several factors likely influence their association, including a history of mental illness prior to a TBI, gender, level of education, severity and type of injury, and time post-injury (Whelan-Goodinson et al., 2010; Perry et al., 2016; Ponsford et al., 2018).

Traumatic brain injury (TBI)-related PTSD has been extensively studied in the military population (Vasterling et al., 2018). As a signature of the conflicts in Iraq and Afghanistan (Okie, 2005), 43.9% of brain-injured soldiers who experienced loss of consciousness met criteria for a PTSD diagnosis (Hoge et al., 2008). Brenner et al. (2010) reported that 26% of troops, returning from Iraq with a diagnosed mild TBI, screened positive for PTSD, compared to 7% without a brain injury (Brenner et al., 2010; Wojcik et al., 2010). Furthermore, a meta-analysis of military and civilian populations found military personnel are nearly 3 times more likely to develop PTSD following a TBI than civilians (Loignon et al., 2020). The target population for these analysis are mostly male dominated as they are, in general, 40% more likely to experience a TBI (Gupte et al., 2019) and are more prominent in the military population.

Trauma-focused therapy such as prolonged exposure therapy (PE) and cognitive processing therapy (CPT) are first-line interventions for individuals who suffer from PTSD (Berg, 2008). However, meta-analyses of randomized-controlled trials (RCTs) (Lewis et al., 2020) and practice-based studies (Herzog et al., 2021) suggest that non-responder rates may be as high as 50%. Fear extinction, a procedure in which the repeated presentation of a conditioned stimulus in the absence of the unconditioned stimulus leads to a decrease in fear expression, is a core mechanism underlying exposure-based therapy, and evidence suggests that PTSD is associated with extinction deficits (Maren, 2001).

Studies have used fear conditioning to examine the impact of TBIs on fear learning in rodents, but far fewer have assessed the effects of TBIs on extinction (Meyer et al., 2012; Genovese et al., 2013; Sierra-Mercado et al., 2015; Davies et al., 2016; Schneider et al., 2016; Hoffman et al., 2019; Corne et al., 2019; Nonaka et al., 2021; Zhao et al., 2021). Within the subset of studies that did examine extinction post-injury, there is variability in outcomes, ranging from no difference in extinction (Sierra-Mercado et al., 2015), to impaired extinction (Zhao et al., 2018) or a resurgence in fear after extinction learning (Corne et al., 2019). There have also been reports of both an increase (Meyer et al., 2012; Schneider et al., 2016; Hoffman et al., 2019) or decrease in freezing during fear acquisition following injury (Genovese et al., 2013; Hoffman et al., 2019; Corne et al., 2019; Nonaka et al., 2021; see Table 1). The lack of consistency in these findings is likely due to several factors including the nature of the brain injury (focal versus diffuse), variations in fear conditioning, extinction, or both, as well as the timepoint after injury at which extinction is assessed. Because TBIs may elicit progressive neurodegeneration throughout the neuroaxis (DeKosky and Asken, 2017), the emergence of extinction deficits (and possibly PTSD) may be critically linked to time post-injury.

**TABLE 1:** Brain-injured rodents show alterations in fear acquisition and extinction.

References	Species	TBI model	% isoflurane	Anesthesia	DPI	Cued	Context	Ext learning	Fear expression	Fear resurgence
Hoffman et al., 2019	Rats *N* = 16–19/group	LFP	2–1%	1×	FC: 2 DPI	Pure tone: 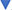 Freezing after TBI White noise: no difference	Pure tone: no difference White noise: 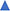 Freezing after TBI	White noise context: 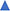 Freezing after TBI	White noise context: 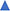 Freezing after TBI	N/A
Zhao et al., 2018	Rats *N* = 8/group	LFP	5–2.5%	1×	FC: 28 DPI	N/A	No difference	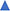 Freezing after TBI	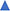 Freezing after TBI	N/A
Meyer et al., 2012	Rats *N* = 10/group	WD	4–3%	1×	FC: 8 DPI	N/A	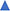 Freezing after TBI	No difference	No difference	N/A
Sierra-Mercado et al., 2015	Mice *N* = 6–12/group	CCI	4–3%	1×	FC: 14 DPI	No difference	No difference	No difference	No difference	N/A
Corne et al., 2019	Mice *N* = 10–15/group	CCI	3–1%	1×	FC: 21 DPI Ext. Resurgence: 42 DPI	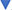 Freezing after TBI	N/A	No difference	No difference	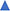 Freezing after TBI
Schneider et al., 2016	Mice *N* = 6–11/group	CCI	5–2.5%	1×	FC: 14 DPI	N/A	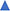 Freezing after TBI	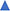 Freezing after TBI	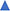 Freezing after TBI	N/A
Nonaka et al., 2021	Mice *N* = 6–11/group	Single and repetitive blast (4×)	3%	1×–4×	FC: 3 DPI 7 DPI 56 DPI	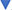 Freezing after TBI (1× and 4×) for trace conditioning at 3 days and 1 week, but not 8 weeks after TBI	No difference	No difference	N/A	N/A
Genovese et al., 2013	Rats *N* = 10/group	Repetitive blast (3×)	5% isoflurane	3×	FC: −1 DPI Ext: 4 DPI–56 DPI	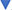 Freezing after TBI	N/A	No difference	No difference	No difference
Davies et al., 2016	Rats *N* = 11–12 per group	WD + Stressor	4–3% isoflurane	1×	FC: 7 DPI	N/A	No difference	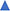 Freezing after TBI, stressed rats and combined treatments	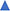 Freezing in only combined treatments	N/A

Summary of fear conditioning and extinction in diffuse and focal models of TBI. LFP, lateral fluid percussion; CCI, controlled cortical impact; WD, weight drop; FC, fear conditioning; Ext, extinction, 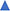 = increased, 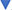 = decreased; N/A, not applicable; DPI, days post-injury; LTM, long- term memory.

Although results vary as to the effect of TBI on extinction, there are substantial individual differences in the response to extinction, even among healthy subjects (Bush et al., 2007; Shumake et al., 2014, 2018). Identifying predictors of extinction would be useful in developing alternative treatments for the non-responders. There is evidence to suggest that individual differences in extinction phenotype are, in part, due to increased orexin neuronal activity in the hypothalamus (Sears et al., 2013; Sharko et al., 2017). Interestingly, these same orexin neurons are activated in the presence of CO_2_ inhalation (Johnson et al., 2011). Indeed, Monfils et al. (2019) found that CO_2_ reactivity predicts extinction phenotypes in rats, likely through the activation of orexin receptors in the lateral hypothalamus. Since CO_2_-exposure is associated with increased activity of orexin neurons in the lateral hypothalamus (Monfils et al., 2019), reactivity to elevated CO_2_ levels may serve as prognostic marker for poor extinction learning. Similarly, those with anxiety disorders display heightened emotional reactivity to a single inhalation of 35% CO_2_ (Telch et al., 2010). In soldiers, CO_2_ reactivity pre-deployment predicted the emergence of PTSD and symptoms of anxiety (but not depression) while deployed in Iraq (Telch et al., 2012). Individuals with PTSD show extinction deficits (Rothbaum and Davis, 2003), reinforcing the potential for CO_2_ reactivity to be a good predictor of extinction phenotypes.

In the present study, we examined brain-injured rats beginning 1 month post-injury (*N* = ∼29/group), a time point at which extinction deficits become evident in rats that received a TBI (Zhao et al., 2018; Corne et al., 2019). We hypothesized that TBI would result in a disruption in extinction, and that CO_2_ reactivity would predict variability in extinction phenotypes.

## 2. Materials and methods

### 2.1. Animals

Adult male Sprague-Dawley rats 60–70 days old (*n* = 88, 300–350 g, Charles River, Raleigh, NC, USA) were tri-housed in transparent polyethylene cages (27 cm × 48 cm × 20 cm) and provided with *ad libitum* food and water. Housing was temperature and humidity-controlled (70°F, 44% humidity) with a 12 h/12 h light/dark cycle. All procedures were approved by the University of Texas at Austin Institutional Animal Care and Use Committee. They were also in accordance with the National Institutes of Health Guide for the Care and Use of Laboratory Animals.

### 2.2. Experiential timeline

**FIGURE 1:**
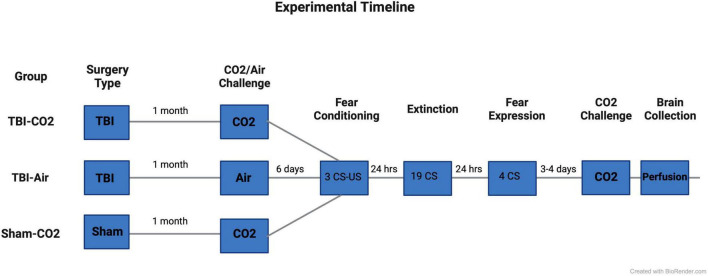
Experimental timeline. Rats first received either a TBI (*n* = 59) or sham (*n* = 29) surgery. 30 days post-injury, half of the TBI (TBI-CO_2_) animals (*n* = 30) and all sham (sham-CO_2_) controls were screened for CO_2_ reactivity. The remaining TBI (TBI-air) animals were screened for normoxic air (*n* = 29). 6 days later, all animals went through fear conditioning, extinction and fear expression separated by 24 h. 3–4 days following fear expression, all animals underwent a final CO_2_ screening and were then sacrificed 1 h later. Created with BioRender.com.

Rats (*n* = 88) underwent either a TBI or sham surgery. At 1 month post-injury (PI), animals that received TBIs were screened for reactivity to CO_2_ (TBI-CO_2_) (*n* = 30) or normoxic air (TBI-air) (*n* = 29), while all sham animals (*n* = 29) were screened for CO_2_ reactivity (sham-CO_2_). Then 6 days later, all groups of rats were fear conditioned using 3 tone shock (US) pairings with conditioned stimulus (CS). The next day, they received an extinction session (19 CSs without US). The day after extinction, rats were tested for fear expression using 4 CSs without US. Either 3 or 4 days later, all animals received a CO_2_ challenge and were sacrificed 1 h later. Brains were removed and prepared for immunohistochemistry (see Figure 1).

### 2.3. Controlled cortical impact

Each rat received a focal brain injury (TBI), produced by a controlled cortical impactor (CCI), as previously described (Igarashi et al., 2007; Semple et al., 2015). Briefly, the rat was anesthetized in a 4% isoflurane chamber and then positioned in a stereotaxic frame with an anesthetic mask delivering 2.5% isoflurane throughout the surgery. A midline incision was made to expose the skull followed by a circular craniectomy midway between bregma and lambda. Each animal was randomly assigned to receive either a TBI (*n* = 59) or sham surgery (*n* = 29). Injury parameters were set at 4.0 m/s velocity and a 2.0 mm depth of penetration using a 6.0 mm convex impactor tip. Sham surgery consisted of the same surgical procedures, including craniectomy, but without CCI. All rats received bupivacaine (0.25%, < 8 mg/kg, subcutaneous) locally at the incision site before craniectomy and buprenorphine (0.05 mg/kg, subcutaneous) immediately following surgery and again 6–8 h later.

### 2.4. Screening for CO_2_ reactivity

**FIGURE 2:**
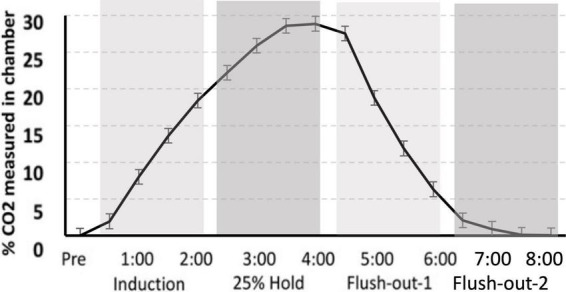
CO_2_ calibration curve. The measurement of CO_2_ in the chamber during “induction,” “hold,” and “flush-out” phases. Over time, CO_2_ was administer into the chamber, held constant around 25% and flushed out with normoxic air. Data is expressed as mean ± standard error (SE).

Gas was delivered through a custom built plexi-glass flow chamber (12″ width × 12″ height × 24″ length). Flow was controlled using a two-stage regulator (Praxair, Inc., Danbury, CT, USA) that delivered gas to the chamber. Ambient air entered the chamber for the first 30 s after the rat was introduced to the chamber. This was followed by a 2 min induction phase, during which 25% CO_2_ was infused into the chamber causing the CO_2_ percentage to slowly rise. CO_2_ was held at 25% for an additional 2 min, after which the chamber was flushed with atmospheric air allowing the CO_2_ percentage to return to normal levels. After 4 min of flushing with atmospheric air, the rat was transferred to its home cage (Figure 2).

### 2.5. CO_2_ behavioral analyses

The scoring system for CO_2_ reactivity was adapted from Monfils et al. (2019). Briefly, each behavior was quantified at baseline (30 s), during CO_2_ induction (2 min), hold period (2 min) and during flush-out period (4 min). Behaviors were monitored through a video camera and were hand scored by an observer, blinded to the experimental condition. The following behaviors were quantified: ambulation (A), grooming (G), rearing (R), and labored breathing (L). For coding purposes, induction was referred to as phase 1, 25% hold phase 2, and the first and second half of flush-out as phases 3 and 4.

### 2.6. CO_2_ challenge and brain collection

At the end of the experiment, all rats received a CO_2_ challenge (as previously described above under CO_2_ screening). One hour post CO_2_ challenge, rats received a lethal dose of Euthasol (Vibric, 1 ml/200 g) and were intracardially perfused with phosphate buffered saline followed by 4% paraformaldehyde (PFA). The brains were extracted and stored in 4% PFA overnight, then transferred into 30% sucrose solution.

### 2.7. Apparatus

All experimental manipulations (fear conditioning, extinction, fear expression) were administered in the same context (operant conditioning chambers; Coulbourn Instruments, Whitehall, PA, USA). Chambers were equipped with stainless-steel rod floor bottoms connected to a shock generator (Model H10-11R-TC-SF; Coulbourn Instruments). All chambers were illuminated under red light. Behavior was recorded by infrared digital cameras (Panasonic, model wvBP344, Osaka, Japan), mounted on the ceiling of each unit. An automated stimulus presentation was elicited using Freezeframe2 software (Coulbourn Instruments, Whitehall, PA, USA). Between each session, chambers were cleaned with Windex (SC Johnson, Racine, WI, USA).

### 2.8. Fear conditioning

Rats were placed in the conditioning chambers for a 3 min habituation period followed by fear conditioning with three 20 s 5 kHz, 80 dB tones conditioned stimulus (CS). Each CS was co-terminated with a 500 ms, 0.7 mA footshock (US). The interval between each CS was on average 120 s in duration. After conditioning, rats remained in the chamber for 3 min and then were returned to the home cage.

### 2.9. Extinction

The day after conditioning, subjects were returned to the same conditioning chambers where they reacclimated for 3 min. This was followed by 19 CS presentations without the US, with variable intervals with a mean of 180 s. After the extinction trial, animals remained in the chamber for 3 min before returning to the homecage.

### 2.10. Fear expression test

The day after extinction, rats were returned to the conditioning chamber, acclimated for 3 min, then presented with 4 CSs without US. The interval between each CS was on average, 120 s in duration. Rats reminded in the chamber for 3 min before returning to the homecage.

### 2.11. Behavioral scoring: freezing

Freezing was defined as the absence of movement aside from breathing, scanning and ear twitching, and excluded sleeping or resting. All behaviors were scored manually by an individual who was blinded to the experimental conditions.

### 2.12. Quantification of lesion volume

Lesion volume, determined at 1 month post-injury, was based upon 40 μm coronal sections stained with hematoxylin and eosin. Measurements of the cortical mantel were taken from both the contralateral and ipsilateral hemispheres using a Nikon Ni-E microscope (Nikon Instruments Inc., NY, USA) spanning Bregma 1.5 to −3.8 mm. This yielded 8–10 sections per brain, using a sampling interval of 12, a 2× objective and a grid size of 400 μm. Cortical measurements were performed by an individual who was blinded to the experimental conditions. Cortical volume was estimated as the product of summed areas of sections and the distance between sections. Lesion volume was then calculated as the difference between volumes of the contralateral and ipsilateral cortices (Tennant et al., 2015; Clark et al., 2019).

### 2.13. Statistical analyses

R (R Development Core Team, 2018, Vienna, Austria), together with the packages beset (Shumake et al., 2018) and nlme (Pinheiro et al., 2017), were used to perform all statistical analyses. Fear acquisition, extinction and fear expression were compared between TBI-CO_2_ and TBI-air rats as well as TBI-CO_2_ and sham-CO_2_ groups using a repeated measures ANOVA, this data included the pre-CS. Data is expressed as mean ± standard error. Exclusion from analysis occurred if video footage was not captured completely (*n* = 7 Extinction).

A modified version of the “best subset” approach to linear regression was used to determine which of the CO_2_-reactivity behaviors accounted for the greatest portion of variance in post-extinction fear expression, the first 2 CS of fear expression, freezing. This approach fits a different linear model for every possible combination of predictor variables. We then used resampling (k-fold cross validation where *k* = 10) to estimate how well each model would predict new samples in terms of mean squared error (MSE). Each model was repeatedly refitted to random subsamples of data and then tested for how well it predicted the remainder of the data. The “best” model was then chosen as the one with the fewest predictors and was within one standard error of the model with the smallest MSE, i.e., the best at predicting new data. Nested cross-validation was used to avoid overly optimistic estimates of prediction error when selecting the best model.

## 3. Results

### 3.1. No differential effects between TBI and sham groups that received CO_2_

**FIGURE 3:**
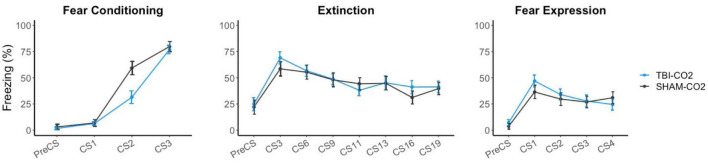
Effects of TBI on fear conditioning, extinction and fear expression 1-month post-injury. There was no difference between TBI-CO_2_ and sham-CO_2_ rats in percent freezing during extinction [F(1, 51) = 0.22, *p* = 0.637] or fear expression [F(1, 56) = 0.16, *p* = 0.68]. TBI-CO_2_ froze less during CS2 during fear acquisition [F(1, 57) = 9.31, *p* = 0.003] but returned to similar freezing rates as sham-CO_2_ rats at the end of fear acquisition and at the beginning of extinction. Data is expressed as mean ± standard error (SE).

Rats received either a TBI (TBI-CO_2_) or sham (sham-CO_2_) surgery followed by a brief exposure to CO_2_ (*n* = 30) or a TBI (TBI-air) with an exposure to normoxic air (*n* = 29), one-month post-surgery, followed by fear conditioning, extinction and fear expression. TBI-air rats served as a control group to ensure there were no interacting effects of surgery and CO_2_ on behavior. We compared groups over the course of fear acquisition, extinction and fear expression (Figure 3). Our primary hypothesis was that TBI would result in a disruption in extinction. We first determined if there was an effect of TBI alone on the measured behaviors. We found no significant differences between TBI-CO_2_ and sham-CO_2_ groups during extinction [F(1, 51) = 0.22, *p* = 0.637] or fear expression [F(1, 57) = 0.114, *p* = 0.736]. However, a significant interaction was found between groups during fear acquisition [F(2, 114) = 6.82, *p* = 0.001] with a main effect between groups [F(1, 57) = 5.30, *p* = 0.02]. This difference seen in fear acquisition is driven by the 2nd conditioned stimulus (CS2) alone and did not persist throughout the remainder of fear conditioning nor did this difference hold up at the beginning of extinction.

### 3.2. Within TBI groups, CO_2_ exposure results in a decreased freezing 24 h post-extinction

**FIGURE 4:**
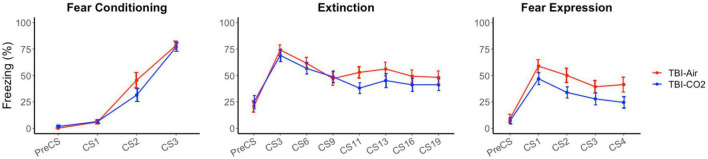
Effect of CO_2_ exposure on fear conditioning, extinction and fear expression 1-month post-injury in rats that received a TBI (TBI-CO_2_). The control group (TBI-air), showed no difference in percent freezing during fear conditioning or extinction than the TBI-CO_2_ group. However, TBI-CO_2_ rats froze less than the TBI-air rats during fear expression [F(1, 57) = 4.01, *p* = 0.05]. Data is expressed as mean ± standard error.

A control group was used to ensure there were no interacting effects of TBI surgery and CO_2_ on preceding behaviors (TBI-air). We compared both groups, TBI-CO_2_ and TBI-air, throughout fear acquisition, extinction and fear expression (Figure 4). There was no significant difference between TBI-CO_2_ and TBI-air groups during both fear acquisition [F(1, 57) = 2.20, *p* = 0.14] and extinction [F(1, 52) = 1.22, *p* = 0.27]. However, TBI-CO_2_ rats showed a decrease in freezing during fear expression compared to TBI-air group [F(1, 57) = 4.01, *p* = 0.05].

### 3.3. CO_2_ reactivity does not predict post-extinction fear expression in rats receiving TBI or sham surgery

We previously showed that CO_2_ reactivity was predictive of post-extinction fear expression behavior in naïve rats (Monfils et al., 2019). Here we tested whether CO_2_ reactivity can predict post-extinction fear expression in injured and sham rats. Post-extinction fear expression was defined as the mean freezing of the first two trials of fear expression. In order to determine if CO_2_ reactivity was a good predictor of post-extinction fear expression, we first ran a regression analysis using the previous *a priori* predictor separately (A3) for the TBI-CO_2_ and sham-CO_2_ groups together and separately. In Monfils et al. (2019), A3 (ambulation during the flush-out phase) had a cross-validation R^2^ estimate of 0.085 meaning it was assessed to be reliably good at predicting 8.5% of fear expression variance. Thus, we considered this an *a priori* predictor. When combining TBI-CO_2_ and sham-CO_2_ rats, A3 did not predict post-extinction fear expression (*t* = 0.076, *p* = 0.939). TBI (*t* = 0.909, *p* = 0.372) and sham groups (*t* = −0.609, *p* = 0.547) alone also showed A3 was also not a significant predictor for post-extinction fear expression. Overall, A3 alone was not a significant predictor of post-extinction fear expression.

In order to examine all of the behaviors measured during the CO_2_ challenge, we analyzed each group (TBI-CO_2_ and sham-CO_2_) separately and together with the best-subset approach. With these parameters, the TBI-CO_2_ and sham-CO_2_ group combined, the null (intercept-only) model was the best model selected for 97% of random subsamples. In the sham-CO_2_ group alone, the best model was also a null model. So, when examining the two groups combined or the sham-CO_2_ group alone, CO_2_ reactivity did not predict post-extinction fear expression.

This same approach was then used for the TBI-CO_2_ group to determine the best predictive effect of CO_2_ reactivity. The null model was selected 50% of the time. Labored breathing during flush-out-1 (L3) also was selected about 30% of the time, and explained 9.3% of the variance in the full sample, but this fell to approximately 0% of the variance in the hold-out samples. Therefore, it seems likely that this predictor is detecting something that is sample specific and is not likely to replicate.

### 3.4. No difference in lesion volume between groups that received a TBI

**FIGURE 5:**
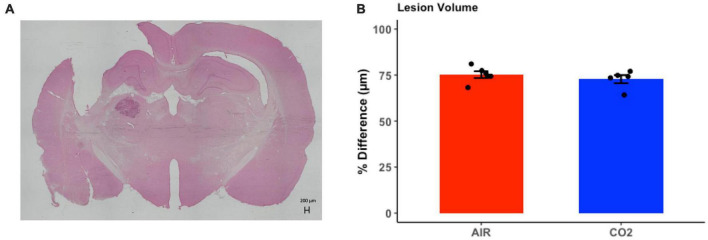
Quantitative assessment of lesion volume at 1-month post-injury in TBI rats. **(A)** Representative H&E stained coronal section, illustrating the site of maximal damage and partial loss of the cortical mantle. **(B)** There were no differences in in lesion volume between groups [t(9) = 0.88, *p* = 0.39]. Data are expressed as mean ± standard error.

Brain injured animals were randomly assigned to 2 groups; namely, those screened for reactivity to CO_2_ (TBI-CO_2_) or normoxic air (TBI-air). Due to differences in freezing behavior, we compared lesion volumes in each of these groups (Figure 5). As this was not part of the original hypothesis, we only chose a subset of each group that upon evaluation had no artifact from brain removal or mounting. There were no significant differences in lesion volume between the group that received CO_2_ and the control group [t(9) = 0.88, *p* = 0.39].

### 3.5. Exploratory analyses

Since this study did not replicate our previous findings, which showed that CO_2_ was a good predictor of post-extinction fear expression in naive rats (Monfils et al., 2019), we next examined what may have been different between the 2 populations. Our aim was to use the naive rats from our 2019 study to compare the distribution of CO_2_ reactivity and freezing during post-extinction fear expression, and the CO_2_ curves between studies.

#### 3.5.1. Shifted distribution in post-extinction fear expression and A3 compared to original naive sample

**FIGURE 6:**
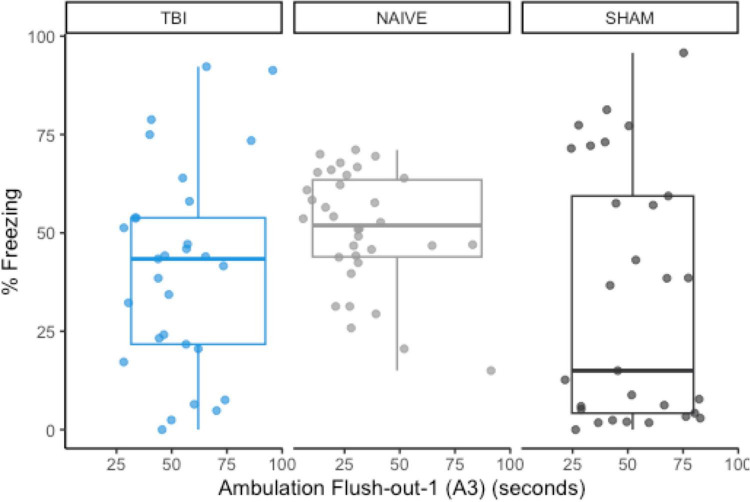
The group distributions for *a priori* predictor A3 and freezing during post-extinction fear expression using data from naïve rats (Monfils et al., 2019). There are noticeable shifts in the distribution of post-extinction fear expression, but A3 remains similarly distributed between groups.

Using previous data from Monfils et al. (2019), we compared the original data distributions of the *a priori* predictor (A3) and post-extinction fear expression to the new distributions of sham-CO_2_ rats. In order for a predictive model to successfully generalize from one sample to another, a minimum requirement would be no large shifts in the observed distributions of either the covariates or the response variables. The observed measurements in this study failed to meet this basic assumption. Compared to the naïve rats in the previous study, post-extinction fear expression freezing was far more variable (SD = 32.0 vs. 14.8) and skewed more toward 0 (*M* = 33.1 vs. 50.7), while the measurement of A3 ambulation was skewed toward higher values (Figure 6).

#### 3.5.2. CO_2_ reactivity is greater in current study during intro and flush out phases

**FIGURE 7:**
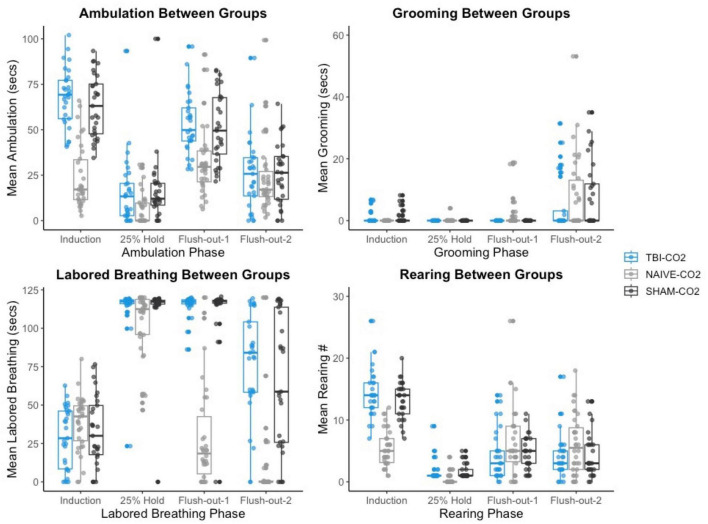
The comparison of measured CO_2_ reactivity between TBI-CO_2_, sham-CO_2_ and naive groups (from Monfils et al., 2019). The TBI-CO_2_ and sham-CO_2_ group both have very similar distributions for all of the behaviors measured. The naive group, during intro and flush-out phases within some behaviors, has on average less measured CO_2_ reactivity.

**FIGURE 8:**
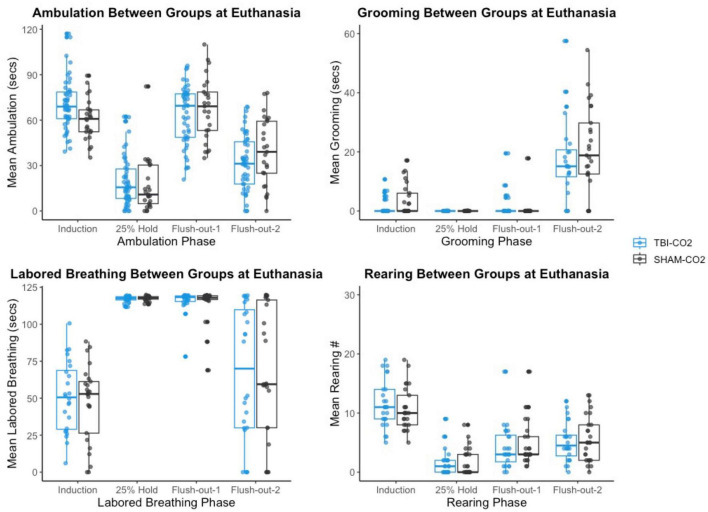
The comparison of measured CO_2_ reactivity between TBI-CO_2_, sham-CO_2_ at euthanasia. The TBI-CO_2_ and sham-CO_2_ group both have very similar distributions for all of the behaviors measured.

We then compared the distributions of CO_2_ reactivity between TBI-CO_2_ and sham-CO_2_ groups, along with the naive sample previously found in Monfils et al. (2019) (Figure 7). An examination of the TBI-CO_2_ and sham-CO_2_ groups, revealed very similar findings for the measured behaviors. This is consistent in both the first CO2 challenge and at euthanasia (Figures 7, 8). The naive group, however, showed visibly lower CO_2_ reactivity during some behaviors, specifically during the induction and flush-out phases of ambulation, rearing and labored breathing. All of these groups showed similar deviation or spread of CO_2_ reactivity, meaning CO_2_ reactivity is defining individual variability similarly but the current study on average visibly displays more CO_2_ reactivity overall.

#### 3.5.3. Compared to the original CO_2_ curve, the induction of CO_2_ is greater and the speed of flush out is slower

**FIGURE 9:**
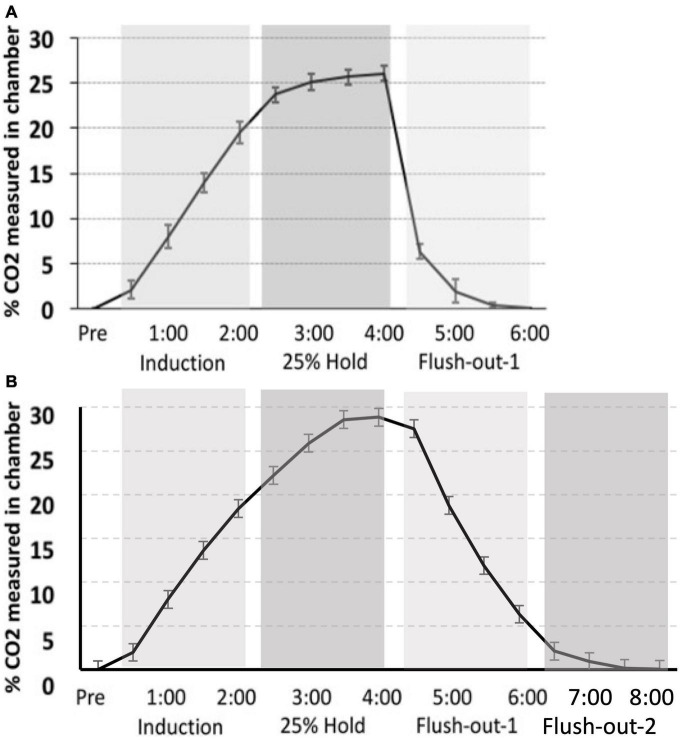
**(A)** Monfils et al. (2019) percent level of CO_2_ in the chamber (CO_2_ curve) as compared to **(B)** the CO_2_ calibration curve for the current study. Although a similar protocol was used, the overall maximum level of CO_2_ is higher in the current study, as well as the slower flush-out time period.

The CO_2_ challenge in this study was meant to replicate that seen in Monfils et al. (2019). However, there is variation in CO_2_ tank flow between the two studies. Despite using the same delivery protocol as we had previously described, there are differences in the actual level of CO_2_ measured in chamber. The hold period in this study peaks at approximately 30% max CO_2_ in the chamber, whereas in our previous study, the hold period peaked around 25%. Thus, CO_2_ in the latter is more rapidly removed during the flush-out phase. These distinctions may have led to differences in CO_2_ reactivity between the two studies (Figure 9).

## 4. Discussion

This study examined the effects of TBI on the extinction of fear and determined if CO_2_ reactivity is a predictor of extinction variability following TBI. Contrary to our *a priori* hypothesis, we found that TBI alone did not have an effect on extinction, but rather the combination of CO_2_ and prior TBI resulted in a decrease in freezing behavior during post-extinction fear expression. We did see a significant decrease in freezing during fear conditioning in TBI rats compared to sham. CO_2_ reactivity did not predict variability seen in post-extinction fear expression in sham or TBI rats. These findings are at odds with our prior hypothesis, but in the context of previous literature, these results have validity.

There are a number of preclinical models of TBI that generate focal and diffuse injuries and are characterized by temporal patterns of neurodegeneration that reflect the type of injury, magnitude, and location of the initial insult (Xiong et al., 2013). As such, it is often difficult to compare behavioral findings across studies where there is inherent variability in behavioral protocols, as well as differences in sample size, the preclinical models employed including when the assays are conducted post-injury. Despite these differences, a few patterns can be extracted from the relevant studies (see Table 1). For example, studies that utilized delayed timepoints (15–28 days) reported extinction deficits in rodents after using either a lateral fluid percussion insult (Zhao et al., 2018) that induces diffuse axonal injury or a CCI (Schneider et al., 2016) that generates a focal cortical injury. These prior studies served as the basis for conducting behavioral analyses at a chronic timepoint where there would be opportunity to compare findings. We chose a CCI model of TBI for this study, because of its well established, reproducible, injury that results in a predictable pattern of neurodegenerative throughout the neuroaxis (Chen et al., 2003; Osier and Dixon, 2016). This model resulted in a decrease in freezing behavior during fear conditioning, which is consistent with another study that used a similar model of TBI (Corne et al., 2019). However, unique to our study, this effect was not sustained for the entire duration of fear conditioning, suggesting a possible delay in fear learning that diminishes over time. Our study likewise examined extinction at a chronic timepoint with a CCI model, however, our findings did not replicate previous work. This may be, in part, attributable to variability in fear conditioning and extinction across protocols.

Previous studies that have examined the effect of TBI on fear acquisition and extinction have all employed somewhat different protocols. Our approach was in line with that used in Monfils et al., 2019. Most previous studies have reported either an increase in freezing or no difference in the injured group relative to sham animals, during extinction and fear expression. However, the present study, as well as others (Hoffman et al., 2019; Nonaka et al., 2021) showed the opposite—a decrease in freezing. One common aspect of the few studies (including our own) that have shown a decrease in freezing after TBI is the repeated use of inhalants throughout the study—in the present case, isoflurane and CO_2_. A repeat blast model of TBI resulted in a decrease in freezing (Nonaka et al., 2021). This model of TBI requires isoflurane exposure up to four times throughout the paradigm. Although groups were not compared directly, sham animals following repeated exposure to isoflurane had overall lower freezing at three days following their last exposure than shams that received only one exposure. Our current study provides a direct comparison between brain-injured rats that have received either isoflurane and CO_2_ or isoflurane and air. The group that had received repeated anesthesia type inhalants also showed a decrease in freezing during fear expression.

The mechanisms that underlie fear conditioning and extinction are well established and are dependent on brain regions that are compromised in individuals suffering from TBI and PTSD. Alterations in the amygdala, hippocampus, thalamus and prefrontal cortext (PFC) result in moderation of fear conditioning and extinction (Maren, 2001). These brain regions are also vulnerable to damage following a TBI (Sato et al., 2001). Reports of fear enhancement during fear conditioning, following injury also reportedly involve increased regulation in N-methyl-D-aspartate (NMDA) receptors in the basolateral amygdala (BLA), along with an overall increase of neurons in the amygdala and a decrease of neurons in the dorsal hippocampus (Meyer et al., 2012; Reger et al., 2012). Extinction impairments following fluid percussion injury coincide with reduced spine density in layers II and III of pyramidal neurons in the hippocampus (Zhao et al., 2018). Although CCI produces focal cortical damage, subcortical regions, including the hippocampus, thalamus and amygdala, also undergo degeneration. Following a CCI, there are decreases in amygdala volume as well as white matter density in the corpus callosum, hippocampus, thalamus and amygdala, which coincide with a resurgence in extinguished fear after successful extinction (Corne et al., 2019). There are also fewer neurons within sub-regions of the hippocampus and changes in volume (Chen et al., 2003; Huang et al., 2021; Knott et al., 2021), as well as reduced GABAergic inhibition in the BLA which overlaps with the development of anxiety-like behavior (Almeida-Suhett et al., 2014). Due to the complex interaction between neurodegeneration and behavior, it is conceivable that a focal cortical injury does is not sufficient to cause the behavioral disruptions reported in diffuse models. However, our results may also have been confounded by the interacting effects of anesthesia (isoflurane) used during surgery and CO_2_.

There is strong evidence that that CO_2_ reactivity may serve as a diagnostic tool in predicting the emergence of fear related disorders. In humans, anxiety disorders display heightened reactivity to a single inhalation of 35% CO_2_ (Telch et al., 2010; Kellner et al., 2018). Similarly, emotional reactivity to 35% CO_2_ is predictive of PTSD and anxiety disorder development following deployment to Iraq in military individuals (Telch et al., 2012). In order to understand possible biological underpinnings, this was modeled in rodents. Similar to humans, rodent studies demonstrate that exposure to moderate concentrations of CO_2_ increase sympathetic activity (Elam et al., 1981), amplify anxiety-like behaviors (Johnson et al., 2011, 2012) and is predictive of extinction phenotypes (Monfils et al., 2019). CO_2_ reactivity accounts for variability found in extinction in healthy adult rats (Monfils et al., 2019). In the present study we did not replicate this finding, even in our sham animals. It bears highlighting that our only sham group for the present study received CO_2_ exposure. A decrease in freezing in rats that received both TBI surgery and CO_2_, suggests that interactions between TBI, CO_2_, and isoflurane interfered with the predictability of CO_2_-reactivity for extinction phenotype.

Indeed, the underlying mechanisms, hypothesized to explain the relationship between CO_2_ and extinction, are known to be affected by isoflurane exposure. Exposure to CO_2_ activates orexin neurons in the lateral hypothalamus (Johnson et al., 2011), which are the same neurons that account for individual differences in extinction (Sharko et al., 2017; Monfils et al., 2019). Isoflurane also inhibits these orexin neurons in the lateral hypothalamus (Kelz et al., 2008). Isoflurane has lingering effects that can induce inflammation and learning impairments up to a month after its use (Cao et al., 2012). Although the exposure to CO_2_ and isoflurane were a month apart, it is possible that cumulative impacts on the same neurons could have affected extinction behavior. A major confound of the repeated blast model of TBI is its repeated use of isoflurane, which may impair fear memory acquisition (Long et al., 2016). CO_2_, which also has the capability to act as a form of anesthesia, may create a confound in interpreting our data. It is possible that together, the repeated exposure of these inhalants could have caused a form of habituation to interoceptive threat that acted via orexinergic neurons in the lateral hypothalamus.

In summary, this study is the first to utilize a chronic, focal model of TBI to examine CO_2_ as a diagnostic tool to explain variability in extinction in the degenerating neuroaxis in male rats. However, the interacting effects of prior TBI surgery, including isoflurane exposure, and CO_2_ have made it difficult to reach definitive conclusions regarding the impacts of TBI on the predictive relationship between CO_2_ reactivity and fear extinction. Recognizing the limitation of studying male rats only, future work should consider comparative studies of both sexes to determine if this interaction between CO_2_ and isoflurane may yield similar interoceptive threat habituation, resulting in better extinction when exposed to both inhalants.

## Data availability statement

The datasets presented in this study can be found in online repositories. All raw data files are available in the Monfils Lab repository, housed in the Texas Data Repository in Dataverse https://dataverse.tdl.org/dataverse/monfilsfearmemorylab. Data is also publicly available at Open Data Commons for Traumatic Brain Injury (ODC-TBI) https://odc-tbi.org.

## Ethics statement

The animal study was reviewed and approved by the University of Texas at Austin Institutional Animal Care and Use Committee and were in accordance with the National Institutes of Health Guide for the Care and Use of Laboratory Animals, and are in line with the ARRIVE guidelines.

## Author contributions

MM, LN-H, and KS designed the study. KS carried out the study, SM, VR, MR, and MD provided technical assistance. JS provided statistical assistance. All authors contributed to the article and approved the submitted version.
